# A case of varicella zoster infection in kidney transplant recipient using immunosuppressant

**DOI:** 10.1002/ccr3.7820

**Published:** 2023-08-24

**Authors:** Abdirahim Ali Nur Adam, Abdulrashid Hashi Mohamed, Mohamed Osman Omar Jeele

**Affiliations:** ^1^ Department of Infectious Disease Mogadishu Somali Turkish Training and Research Hospital Mogadishu Somalia; ^2^ Department of Internal Medicine Mogadishu Somali Turkish Training and Research Hospital Mogadishu Somalia

**Keywords:** immunosuppressant, kidney transplant recipient, Somalia, varicella zoster infection

## Abstract

Kidney transplant recipients must take lifelong immunosuppression to prevent acute or chronic allograft injury. However, they are also at risk for opportunistic infections due to compromised immune cell functionality. Disseminated HZ in kidney transplant recipients can result in a very high overall mortality rate of up to 30%. Here we described a 23‐year‐old male patient who presented to the emergency room with a complaint of high‐grade fever, chills, and non‐dermatomal lesion of varicella zoster skin infection that affected the face and trunk. After investigation the patient was diagnosed with chickenpox clinically and was managed with complete recovery and early hospital discharge.

## Graphical abstract

Admission pictures of the patient.

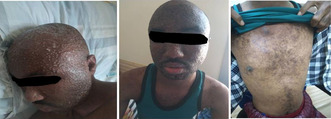

## 1 INTRODUCTION

Kidney transplant recipients are needed to take immunosuppressive for the rest of their lives to prevent acute or chronic allograft rejection. However, their compromised immune system put them at risk for opportunistic infections as well.^1^ Infection with the varicella zoster virus (VZV) is an unusual in recipients of kidney transplants at a frequency rate from 1% to 11%, but the severity is more significant in transplant patients compared to the overall population.^2^, ^3^ Fever and a self‐limiting rash on the skin and occasionally the mucosa may be the present symptoms of varicella infection. Additionally observed symptoms include headache, malaise, and appetite loss. The rash starts off as macules, quickly develops into papules, and then goes through a vesicular stage and crusts over the lesions. After 1–2 weeks, crusts flake off.^4^ Disseminated herpes zoster (HZ) in kidney transplant recipients can result in a very high overall mortality rate of up to 30%.^5^

Here we describe a 23‐year‐old male patient with a history renal transplant who presented to the emergency department with complaint of high‐grade fever, chills, and generalized rash for 5 days.

## 2 CASE PRESENTATION

A 23‐year‐old male patient presented to the emergency department with a complaint of high‐grade fever, chills, and generalized rash for 5 days. The patient was diagnosed with the end‐stage renal disease 4 years ago which he was put on routine hemodialysis program for 1 year. He underwent renal transplant 3 years ago and since then he did not needed any hemodialysis sessions. Following his renal transplant surgery the patient was put under immunosuppressants drugs (mycophenolate mofetil 2 g/day, prednisolone 20 mg/day, and tacrolimus 12 mg/day). The patient did not mention any increase in the dosage. He had no known history of chickenpox at young age and no history of diabetes. He never had any vaccination prior to his renal transplantation including varicella vaccine. There was no history of contact with chickenpox patient.

**FIGURE 1:**
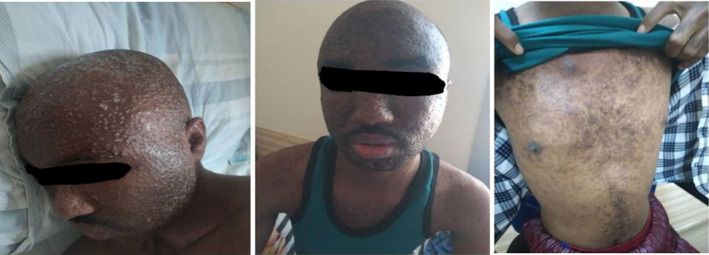
Widespread erythematous papules and vesicles in the face and trunk on admission.

On presentation, the patient was fully conscious, alert, and cooperative and with the following vital signs: BP 145/90 mmHg, pulse 90 times per minute, RR 14 bpm, and temperature 38.6°C. On examination, there was no jaundice or pallor in the conjunctiva. He had no palpable lymph nodes, discomfort, or stiffness in the neck. The rashes were variously healed widespread erythematous papules and vesicles in the face and trunk (Figure 1). Chest examination was within normal limit with no rales or wheezing. He had normal bowel sounds on abdominal examination with no organomegaly. Limb examination showed adequate turgor and no edema, inguinal lymph nodes were not palpable. Blood tests revealed a hemoglobin (Hb) level of 16.6 g/dL (12–17 g/dL), hematocrit (Ht) 49.8% (42%–52%), leukocyte 7.60 × 1000/mm^3^ (4–10 × 1000/mm^3^), platelet 200 × 1000/mm^3^ (×1000/mm^3^), MCV 87.9 fL (80–100 fL), MCH 29.4 pg (27–34 pg/cell), MCHC 33.4 (32–36 g/dL), creatinine 0.82 mg/dL (0.6–1.35 mg/dL), urea 34 mg/dL (10–45 mg/dL), random blood glucose (RBS) 110 mg/dL (60–110 mg/dL), sodium 142 mEq/L (135–145 mEq/L), potassium 3.84 mEq/L (3.5–5.5 mEq/L). Urine examination was unremarkable. Abdominal ultrasonography only revealed bilateral atrophic kidneys and transplanted kidney in the pelvic region.

The patient was admitted to infectious disease department under the diagnosis of chickenpox infection using clinical assessment. He was started with intravenous acyclovir 3 × 750 mg for 5 days which was later switched to oral acyclovir 5 × 800 mg and acyclovir lotion and antipyretics.

**FIGURE 2:**
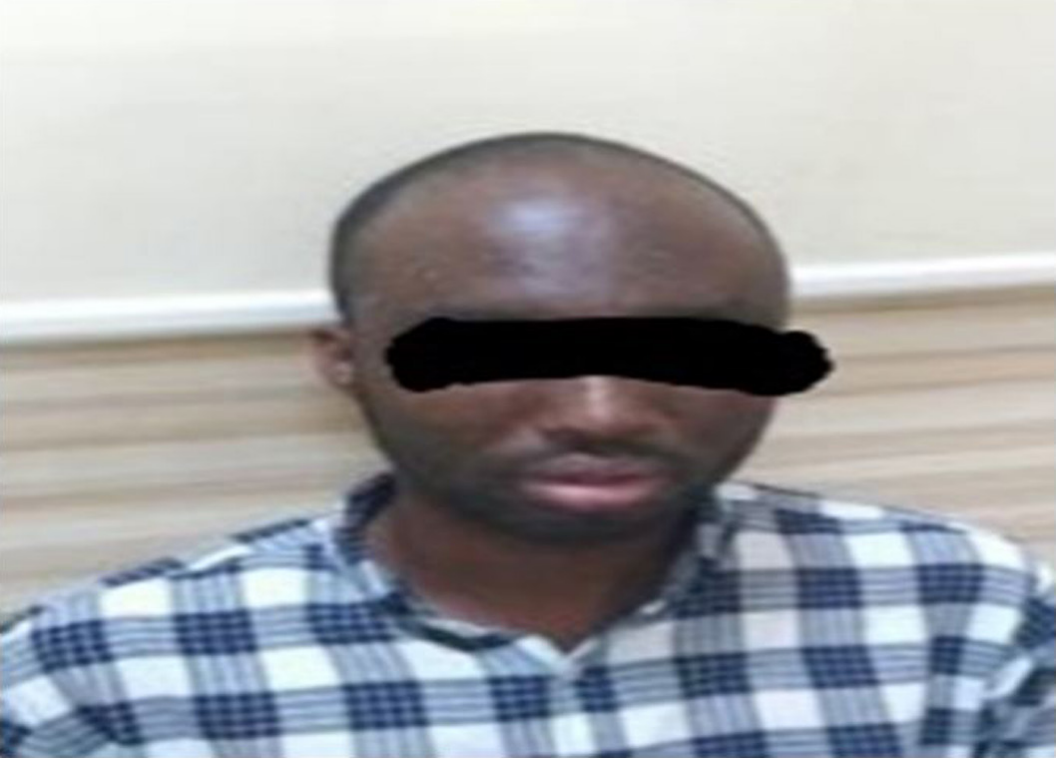
After 3 weeks follow‐up drastically improved rashes.

As his rash started subsiding with normal temperature he was discharged to home with close monitoring. After 3 weeks, the patient came for follow‐up, his rashes had drastically improved and had no fever or chills (Figure 2).

## 3 DISCUSSION

Kidney transplantation is a therapeutic choice for end‐stage renal disease. Kidney transplantation offers the patients a better quality of life as they are free from fluid and potassium restriction, better metabolic function, and normal hemoglobin as normal kidney function return.^6^ Immunosuppressants are essential for the allograft function to be successful. These medications functions by reducing the allograft rejection response and are used for maintenance, treating rejection reactions, and induction (strong immunosuppression in the early days after transplantation). However, using immunosuppressants also has drawbacks.^6^ The body will be more vulnerable to a variety of opportunistic infections due to the body's immune system being suppressed, which can both jeopardize the outcome of kidney transplants and result in fatalities.^7^, ^8^ Up to 44.9%–82% of recipients of kidney transplants get infections postoperatively, including viral infections like cytomegalovirus and varicella as well as urinary tract infections and pneumonia. The highest percentages of infections among patients who are on immunosuppressants are caused by bacteria, fungi, and viruses in 50%, 30%, and 5%, respectively; while 15% of infections are considered as polymicrobial.^9^ Fever, pneumonia, enteritis, meningitis, and encephalitis are some of the clinical manifestations of viral infections. As a result, the immune system is suppressed, which raises the risk of opportunistic infections.^10^

For the past 3 years, our patient has been living with a transplanted kidney and during that time there have been no complaints. He was using corticosteroids, mycophenolate mofetil, and tacrolimus as his three immunosuppressants.

Rapid diagnosis is necessary to select the appropriate antiviral therapy in immunocompromised patients at the time of acute VZV infection. Tissue culture can be used to isolate VZV; this technique is not, however, quick enough to affect therapeutic decision‐making. The virological assay to identify VZV antigens using monoclonal antibodies is the quickest test. Due to a lack of resources, neither culture isolation nor any other techniques were used to diagnose this patient. The clinical signs actually showed varicella infection (chickenpox).^11^

Giving an antiviral medication as soon as possible, preferably within the first 24 h after the rash initially starts, will produce the best results. Antiviral medication can be substituted orally if clinical improvement occurs.^12^

Although being the high‐risk group for infections, vaccinations had never been administered to our patient before. Some recommendations call for the delivery of vaccines to every patient receiving an organ transplant. Seronegative individuals should receive two doses of pretransplant immunization, spaced 4 weeks apart, to help prevent serious infection. The chosen vaccine must be suitable for chronic kidney disease patients.^13^

## 4 CONCLUSIONS

Patients undergoing kidney transplants frequently have immunosuppressive conditions, making them more susceptible to infections like severe VZV infection. Prompt diagnosis and early initiation of antiviral therapy are crucial. The VZV vaccine prior to the transplantation is required to protect the kidney transplant patient from infection. Antiviral medication should be taken into consideration if kidney transplant recipients have a serious varicella infection. Close monitoring and timely intervention are essential in managing VZV infections in this vulnerable population.

## AUTHOR CONTRIBUTIONS

**Abdirahim Ali Nur Adam:** Conceptualization; methodology; writing – original draft. **Abdulrashid Hashi Mohamed:** Writing – original draft. **Mohamed Osman Omar Jeele:** Conceptualization; supervision; writing – review and editing.

## FUNDING INFORMATION

The authors declare no funding source for this research.

## CONFLICT OF INTEREST STATEMENT

The authors declare no competing interests in relation to this study.

## ETHICS STATEMENT

Mogadishu‐Somali Turkish Training and Research Hospital ethics committee waived approval for this case report.

## CONSENT

Written informed consent was obtained from the patient to publish the details and the images in this case report anonymously.

## Data Availability

The data are available for the corresponding author ifrequested.
